# Effectiveness of Continuous Positive Airway Pressure in Treating Hypertension in Obstructive Sleep Apnea: A Traditional Review

**DOI:** 10.7759/cureus.42111

**Published:** 2023-07-19

**Authors:** Naisargi Shrikant Modi, Parth S Bajoria, Prathma Anandbhai Dave, Ralph Kingsford Rohit, Charu Tibrewal, Priyansh Patel, Siddharth Kamal Gandhi, Sai Dheeraj Gutlapalli, Keith Diaz, Jay Nfonoyim

**Affiliations:** 1 Department of Internal Medicine, Civil Hospital Ahmedabad, Ahmedabad, IND; 2 Department of Internal Medicine, GMERS (Gujarat Medical Education and Research Society) Medical College Gandhinagar, Gandhinagar, IND; 3 Department of Internal Medicine, Medical College Baroda, Vadodara, IND; 4 Department of Internal Medicine, Dayanand Medical College and Hospital, Ludhiana, IND; 5 Department of Internal Medicine, California Institute of Behavioral Neurosciences & Psychology, Fairfield, USA; 6 Department of Internal Medicine, Shri M.P. Shah Government Medical College, Jamnagar, IND; 7 Department of Internal Medicine, Richmond University Medical Center, New York City, USA; 8 Department of Pulmonary and Critical Care Medicine, Richmond University Medical Center, New York City, USA

**Keywords:** hypertension, clinical effectiveness, continuous positive airway pressure (cpap), osa, obstructive sleep apnoea

## Abstract

Almost one billion individuals worldwide suffer from obstructive sleep apnea (OSA). The most widely used treatment for OSA has been continuous positive airway pressure (CPAP), but its effect on blood pressure (BP) has been challenged. Our review aims to evaluate the effects of treating OSA with CPAP on BP and BP-related morbidities in adult hypertensive patients. Medical subject headings (MeSH) terminology was used to search the PubMed Central, MEDLINE, and PubMed databases for articles on the use of CPAP in OSA patients with hypertension. We selected various forms of academic writing, encompassing complete texts that were published in the English language. The study included a total of 21 papers. OSA is a serious health concern associated with a higher risk of cardiovascular disease, kidney disease, pulmonary hypertension, and aortic stiffness, which is brought on by the periodic hypoxia caused by nocturnal respiratory episodes. For individuals with moderate-to-severe OSA, CPAP therapy has been shown to have a considerable long-term benefit with a median drop of 11 mm Hg, and high adherence results in a decrease in diastolic BP. CPAP therapy directly lowers BP in OSA patients with a body mass index (BMI) of more than 30 kg/m2 and has also demonstrated improvement in early signs of atherosclerosis with lower nocturnal systolic BP levels. OSA patients with resistant hypertension also experienced lower BP after using CPAP for a year. Therefore, our findings suggest that obesity, hypersomnolence, high nocturnal BP, prolonged CPAP usage, and resistant hypertension may all have a major impact on the BP response to CPAP therapy in individuals with severe OSA.

## Introduction and background

Obstructive sleep apnea (OSA) affects almost one billion people worldwide. To minimize adverse health effects and maximize cost-effectiveness, better diagnosis and treatment procedures are required [1]. Men and women in their middle years who fit the diagnostic criteria for OSA are estimated to be 34% and 17%, respectively [2]. Repeated upper airway collapse during sleep, a feature of OSA, can result in sleep fragmentation, oxygen desaturation, autonomic dysfunction, and excessive daytime drowsiness [3]. The immediate and long-term physiological stressors brought on by persistent upper airway blockage can lower a person's quality of life while also increasing cardiovascular (CV) morbidity and death. In actuality, OSA is thought to affect more than 40% of people with CV disease, making it a very common condition [3]. The apnea-hypopnea index (AHI), which quantifies the number of apnea and hypopnea incidents occurring per hour of sleep, is utilized to further classify OSA. As a result, OSA can range from mild (AHI: 5-15), moderate (AHI: 15-30) to severe (AHI: >30) [4]. For more than 30 years, continuous positive airway pressure (CPAP) has been the initial and most popular treatment for OSA. It is the continuous application of positive airway pressure that maintains the caliber of the upper airway, acts as a pneumatic splint to keep it open, and raises lung volume to give tracheal traction [5]. Hypertension (HTN) is characterized by an increase in systolic and diastolic arterial blood pressure (BP) above a predetermined threshold of 140 mmHg and 90 mmHg, respectively [1]. BP refers to the perpendicular pressure exerted by blood on the walls of blood vessels. Based on its underlying cause, HTN can be categorized as either primary or secondary. The risk factors include kidney disorders, diabetes, a sedentary lifestyle, older age, high-salt diets, and obesity [6]. As an estimated 50% of OSA patients have HTN and 30% of hypertensive individuals also have OSA, HTN and OSA frequently coexist in patients [3]. For a significant number of years, there has been considerable interest in the connection between OSA and HTN. Research evaluating the impact of OSA treatment on BP, however, has been more debatable, with the majority of research revealing very modest changes in BP following the administration of nasal CPAP [7]. The objective of this literature review is to evaluate the effects of treating OSA with CPAP on BP levels and associated complications related to high BP in individuals with HTN.

Methodology

In conjunction with all authors, PubMed Central, MEDLINE, and PubMed databases were searched in May 2023 using various combinations of continuous positive airway pressure, hypertension, and obstructive sleep apnea. A total of 1120 papers were identified, and a free full-text filter was applied to yield 420 papers. However, the following search strategy was selected based on the medical subject headings (MeSH) vocabulary: ("Continuous Positive Airway Pressure" [Majr]) AND ("Hypertension/prevention and control" [Majr] OR "Hypertension/therapy" [Majr]) AND ("Sleep Apnea, Obstructive/complications" [Majr] OR "Sleep Apnea, Obstructive/prevention and control" [Majr] OR "Sleep, Obstructive/prevention and control" [Majr]). No time limits were set. We selected all types of study literature that was published in English with full text. Articles were excluded where the free full text could not be retrieved. Duplicate publications and gray literature were also excluded. All articles underwent a screening process, and any discrepancies or differences in opinions among the authors were resolved through discussion until a consensus was reached. After discussion among all the authors, a total of 21 studies were unanimously included in the review. Of those 21 studies, 15 were review articles, four were randomized controlled trials (RCTs), and two were meta-analyses.

## Review

OSA is characterized by snoring, daytime sleepiness, restless sleep, morning headaches, asthenia, asphyxiant arousal, performance changes, and the occurrence of obstructive airway events during sleep. Additionally, OSA can be diagnosed in the absence of sleep-related symptoms if there are more than or equal to 15 airway obstruction episodes per hour of sleep [8]. The disease is distinguished by recurring blockages in the upper airway, leading to episodes of sleep apnea and intermittent periods of reduced oxygen levels (hypoxia). OSA affects approximately 14% of males and 7% of females in the entire adult population [8]. One of the major CV diseases, HTN, is associated with OSA [9]. It is often an undiagnosed clinical condition in hypertensive patients. In fact, over 70% of patients with resistant HTN have OSA [10]. The American College of Physicians advises using CPAP as the first treatment for OSA to keep the airway open [11]. It has proved to be the most effective treatment for OSA and has had a positive impact on patient’s BP without requiring any modification to their anti-hypertensive medication, according to the study [12].

Pathophysiology

**Figure 1:**
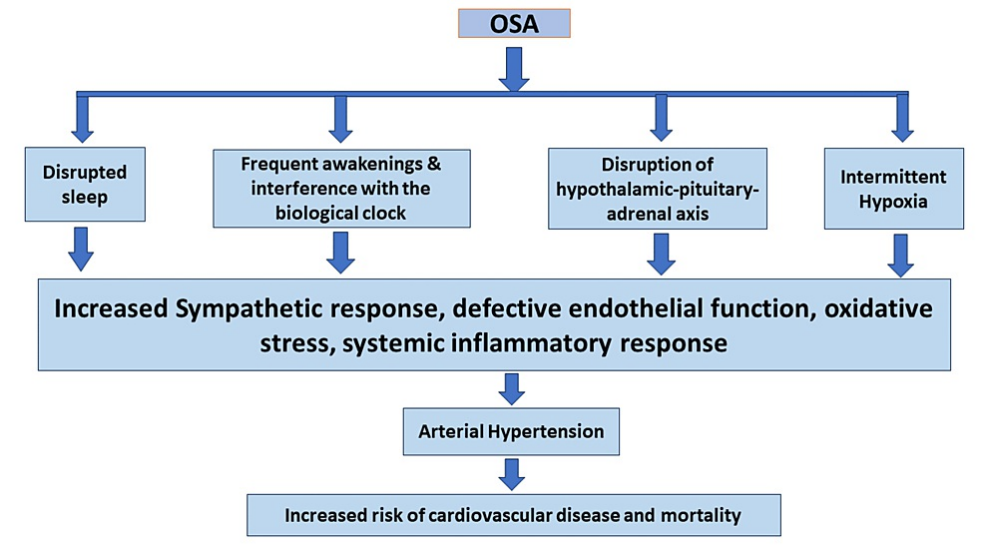
Mechanism of OSA and increased cardiovascular mortality. OSA: obstructive sleep apnea Image credit: Naisargi Shrikant Modi, Priyansh Patel, and Charu Tibrewal

The mechanisms leading to vascular damage in OSA are not well defined, although it is hypothesized that the cycles of intermittent hypoxia generated by nocturnal respiratory episodes lead to altered chemoceptor and baroceptor activity [8]. At the same time, hypoxia generates a range of neurophysiological and biochemical alterations that disrupt the central neuronal regulation of BP [13]. This enhanced activation of the sympathetic nervous system, together with endothelial dysfunction, oxidative stress, and inflammation, has been believed to play essential roles in the link between OSA and HTN [11]. Nocturnal hypoxia also directly elevates peripheral arterial smooth muscle tone, leading to persistent BP elevation [13]. Another substantial evidence has illustrated the relationship of OSA with hyperaldosteronism in patients with resistant HTN. It is theorized that aldosterone excess causes fluid accumulation in the neck and hence leads to higher upper airway resistance, which may worsen the severity of OSA and the corresponding increase in BP levels [11]. Recurrent episodes of hypoxemia and re-oxygenation are known to generate oxidative stress, inflammation, and activation of coagulation with decreased vascular endothelial function. Earlier symptoms of atherosclerosis, such as increased intima-media thickness, may be present in the population with obstructive airway events. Furthermore, evidence from the literature suggests that patients with OSA had greater serum levels of vasoactive mediators, such as endothelin, and low levels of nitric oxide endothelial synthase, which would contribute to decreased flow-mediated dilation of the vasculature [8]. Moreover, one study demonstrates intermittent hypoxia as the key driver for CV and metabolic alterations [14]. Sympathetic activation, vascular endothelial damage, metabolic dysregulation, oxidative stress, and inflammation may lead to elevated BP with a pathological circadian profile and fluctuation. In fact, investigations have indicated that patients with OSA display vasoreactive dysfunction, vascular remodeling, and a faster progression of atherosclerosis [14]. Pharyngeal collapse causes OSA to cause intermittent hypoxemia and carbon dioxide (CO2) retention, with oxygen saturation falling as low as 60% in severe cases. As a result, there are disrupted autonomic and hemodynamic responses during sleep, which are particularly characterized by sympathetic activity mediated by chemoreflexes and the influence of peripheral blood vessel constriction, leading to elevated BP throughout the night. Furthermore, the increase in BP can be attributed to various factors, including recurrent hypoxemia, the presence of vasoactive substances such as endothelin and angiotensin, as well as other pathological mechanisms such as oxidative stress, systemic inflammation, and insulin resistance [10]. A number of studies have demonstrated that CPAP decreases sympathetic activity, decreases the formation of free oxygen radicals and several circulating inflammatory markers, and restores the endothelial dysfunction linked to OSA [15]. Figure 1 describes the mechanism of OSA with increased CV mortality.

Mechanism of BP reduction with CPAP

In patients with sleep apnea, CPAP therapy enhances endothelial function, decreases abnormally elevated levels of circulating apoptotic endothelial cells, reduces neutrophil and monocyte production of free radicals, attenuates C-reactive protein (CRP), increases vasodilator levels, and mediates a decrease in vasoconstrictor levels [16]. It is possible that intricate pathways, which have not yet been fully understood, mediate some of the cardiovascular effects of sleep apnea and lead to resistant HTN; CPAP therapy lessens these effects by a mechanism different from that of medication [16]. It is important to note here that CPAP therapy has been linked to decreased renin-angiotensin system activity and decreased plasma aldosterone. Furthermore, CPAP decreases sympathetic nervous activity by lessening sleep disturbance [17]. Adherence to CPAP therapy may also alter the plasma levels of peptides and hormones linked to CV function as well as the microRNA (miRNA) profiles [17]. Randomized studies have repeatedly shown that CPAP therapy for OSA reduces BP by two to three mmHg. This effect would be anticipated to lower CV events by 5% to 10%, making it clinically important. Some studies have hypothesized that a more significant effect could develop over time to allow for vascular remodeling [18]. In addition, CPAP therapy has a number of potential BP-lowering mechanisms. The airway is held open by CPAP, which prevents collapsing and improves breathing. This suppresses vascular endothelial dysfunction by stabilizing oxygenation and lowering oxidative stress [19]. Moreover, patients with systemic HTN have higher levels of aldosterone secretion because repeated apneic episodes may result in increased aldosterone and renin secretion. This could affect fluid balance and BP levels in OSA. [19]. After eight weeks of treatment, individuals with resistant HTN who used CPAP therapy experienced a decrease in systolic BP (SBP) and, to a lesser extent, diastolic BP (DBP) [15]. Patients with symptomatic or resistant HTN, for example, benefit significantly from CPAP therapy. The results of the CPAP treatment were observed to be impacted by nocturnal HTN. In contrast to nocturnal normotensive subjects, nocturnal hypertensive patients showed a beneficial shift in BP following CPAP treatment (decreased night-time BP values) [20]. The preferred course of treatment for patients with moderate to severe obstructive sleep apnea-hypopnea (OSAH) symptoms is the delivery of CPAP while they sleep. It has been demonstrated that all of the aforementioned "intermediate mechanisms" that cause or exacerbate systemic arterial HTN (SAH) in OSAH can be favorably modified by CPAP [21].

Complications

OSA is one of the main causes of secondary resistant HTN, with an extremely high prevalence of 70%-83% in the population. It is linked to the emergence of arterial hypertension and abnormalities in the physiological circadian variation of BP control [8]. When the recommended BP goals cannot be met despite lifestyle changes and combination therapy with at least three different anti-hypertensive drug classes, including a diuretic, taken at the maximum recommended or tolerated dose, a patient is said to have "resistant" (or "refractory") HTN. Growing evidence from the literature demonstrates that OSA is linked to increased CV and renal morbidity and that OSA is a separate risk factor for the emergence of arterial HTN [8]. As a result of chronic intermittent hypoxemia and a higher sensitivity to hypoxic pulmonary vasoconstriction, OSA may also contribute to high-altitude pulmonary edema [22]. The most prevalent kind of sleep-disordered breathing, OSA, contributes to the pathophysiology of atherosclerosis, congestive heart failure, systemic HTN, and arrhythmias. Without any underlying lung or heart conditions, about 20% of OSA patients experience moderate pulmonary HTN [23]. According to Alchanatis et al., the development of pulmonary HTN is associated with advanced age, a higher body mass index (BMI), and a reduced partial pressure of oxygen in the arterial blood (PaO2) during wakefulness. OSA patients typically have mild pulmonary HTN. Pulmonary artery pressure in 42 pulmonary HTN patients examined by Laks et al. ranged from 20 to 52 mmHg, with an average value of 29 mmHg [23]. Moreover, there is a correlation between OSA and increased aortic stiffness, which can be assessed through the measurement of carotid-femoral pulse wave velocity (cfPWV) [24]. According to traditional risk factors, the 10-year CV risk for the average patient with OSA is 30% (risk of myocardial infarction and stroke combined) [25]. Due to the significant role that HTN plays in both fatal and nonfatal CV disorders, it is imperative that the causes and methods for managing them are promptly identified and implemented. The maintenance of a healthy sleep-wake cycle significantly affects a wide range of neuroendocrine systems, all of which are crucial to maintaining homeostasis in the CV and metabolic systems [26]. Sleep disorders and excessive daytime sleepiness have a significant economic and social impact that fully justifies their inclusion as public health concerns due to the financial burden that all of these diseases place on the national health system, the risks of workplace accidents and traffic accidents, and, finally, the losses due to reduced productivity [26]. One study found that CPAP therapy significantly reduces the above-mentioned events along with reducing the renal resistive index (RRI) [8]. Of the 84 miRNAs examined, changes in the expression of 47 of them were connected to adherence to CPAP use. Significant differences were observed in the changes in miRNA profiles between responders and non-responders [19]. Responders showed a general drop in the expression levels of circulating miRNAs that target the cardiovascular system after CPAP treatment, whereas non-responders showed no change or even an increase in the expression levels of several of the circulating miRNAs [19]. According to extensive prospective studies, a 3.3 mmHg drop in BP should be linked to a 20% reduction in the risk of stroke and a 15% reduction in the risk of coronary heart disease events [25].

Qualitative analysis of clinical studies for the effectiveness of CPAP

The HIPARCO project is a high-quality clinical trial that investigates the specific extent to which CPAP reduces BP in patients with both OSA and resistant HTN [18]. The researchers confirm the significant frequency of OSA, roughly 89%, in a cohort with resistant HTN. CPAP therapy may offer a significantly better risk-benefit profile compared to using a fourth- or fifth-line antihypertensive drug for individuals who do not respond to standard antihypertensive medications [18]. Budhiraja et al. showed that BP decreased with both therapeutic CPAP and a placebo (CPAP delivered at an ineffective pressure). In a different study, only patients with daytime sleepiness who have sleep apnea demonstrated improvement in HTN with CPAP use. Therefore, it is yet unknown whether CPAP lowers blood pressure and in which subgroup of OSA patients [16].

Factors Affecting The Effectiveness of CPAP

CPAP adherence: After multivariate adjustment, Shirahama et al. discovered a significant difference in diastolic BP trajectories in a sizable sample of OSA patients when comparing patients with strong or poor CPAP adherence across the 24-month follow-up period [17]. A significant reduction in DBP was observed in the patient group with good CPAP adherence (a mean usage rate of CPAP for more than four hours, 70% derived from each point) during the follow-up period, showing a considerable long-term benefit of CPAP therapy [17]. At the end of the follow-up, good adherence was defined as an average cumulative usage of CPAP of more than four hours per night. According to Campos-Rodriguez et al., in individuals with resistant HTN and OSA, good CPAP adherence is an achievable and practical aim. Short-term adherence and prior strokes both predicted long-term adherence [27].

Obesity: With a mean BMI of less than 30 kg/m2, Shirahama et al. specifically planned their study to examine the long-term effects of CPAP therapy on BP in a cohort of relatively slim OSA patients. According to the World Health Organization (WHO), a BMI equal to or greater than 30 kg/m2 is classified as obese [17]. Because obese people have sympathetic hypertonia as a result of increased insulin secretion, they may respond to good CPAP compliance more strongly than relatively thin populations in terms of decreasing BP. However, in both the groups with good and poor CPAP adherence, the body weight tended to rise throughout the course of the 24-month monitoring period. This shows that CPAP therapy directly lowers BP without the need for weight loss [17].

Symptomatic OSA: In a study conducted by Robinson et al., it was observed that in patients with moderate to severe OSA who did not experience excessive daytime sleepiness, the use of therapeutic CPAP did not result in significant differences in the primary outcome measure, which was the mean 24-hour ambulatory BP [25]. SBP and DBP did not alter throughout wakefulness or sleep, according to further investigation. In contrast, the BP drops in hyper somnolent OSA patients receiving CPAP treatment. Additionally, it raises the possibility that hypersomnolence may have a role in the HTN caused by sleep apnea [25]. The most recent data aligns with the findings of Barbe et al., who also did not observe a decrease in BP with therapeutic CPAP compared to subtherapeutic CPAP after six weeks of treatment, as assessed by 24-hour ambulatory BP measurements [25]. As expected based on the existing literature regarding patients with hypersomnolence, this study focused on hypertensive individuals with OSA but without daytime hypersomnolence and did not demonstrate a significant reduction in BP with CPAP therapy [25]. This shows that hypersomnolence and human sleep apnea are both caused by sleep fragmentation, which may also play a role in the etiology of HTN. Thus, as suggested by Barbe et al., it is not advisable to treat non-hyper somnolent patients with OSA in order to reduce their risk of CV disease [25].

Diurnal variations in BP: In their meta-analysis, Hue et al. discovered that CPAP had significant BP-lowering effects, primarily on nocturnal SBP, but that its effects on diurnal SBP appeared to be less dramatic [10]. On the contrary, according to research by Dernaika et al., using CPAP therapy was associated with a decrease in daytime BP in patients with resistant HTN; this decrease became noticeable six months following the start of treatment and persisted for 12 months [10]. When using CPAP to treat 12 hypertensive OSAH patients, Bottini et al. discovered that nighttime SAH was quickly reduced, but daytime SAH dropped considerably only after six months [21].

Duration of CPAP use: Shirahama et al.'s trial mentions that long-term (24-month) CPAP use is associated with decreased SBP and DBP in CPAP users. The study also mentions that those who adhered well to their CPAP regimen showed a considerable reduction in DBP [17]. The above finding is further supported by a study performed by Sánchez-de-la-Torre et al., which reveals that consistent CPAP use lowers 24-hour mean BP by a median of 4.5 mmHg. Moreover, the study revealed that participants experienced a median reduction in BP of 11 mmHg, and this decrease was significantly associated with a lower relative risk for several CV outcomes, including stroke, coronary heart disease, heart failure, major CV events, CV death, and overall mortality [19]. In contrast to that, Sekizuka et al. showed that even two-week CPAP therapy can lower BP in OSA patients. It also mentions that even three-day CPAP therapy lowers diurnal and nocturnal BP and causes OSA patients to transition from non-dippers to dippers. Furthermore, three-month CPAP treatment decreased 24-hour SBP (9.71 mmHg), 24-hour DBP (6.98 mmHg), and daytime DBP (6.12 mmHg) in OSA patients with ambulatory BP monitoring (ABPM) confirmed resistant HTN [19]. According to Bottini et al., the length of CPAP therapy is crucial to observe any effects on OSAH patients' arterial BP. This is consistent with the idea that individuals with OSAH may require more than a few months of CPAP therapy to see a reduction in sympathetic activity [21].

Severity of OSA: Castro-Grattoni et al. discovered that there was a decrease in erythrocyte sodium sensitivity (ESS), red blood cells, hemoglobin, and norepinephrine urine levels after six months of CPAP treatment, indicating a decrease in sympathetic activity. However, neither the 24-hour BP nor the nighttime BP showed any discernible changes [20]. However, Robinson et al. demonstrated in randomized controlled trials that CPAP therapy lowers 24-hour BP in patients with more severe illnesses [25]. Further, Lai et al. showed that CPAP therapy for six months significantly lowers clinic BP and the 24-hour mean SBP and DBP values among patients with OSA-related resistant HTN. The substantial improvement in intima-media thickness (IMT) and renal resistive index (RRI) indicates that CPAP therapy improves endothelial dysfunction and early indicators of atherosclerosis [8]. In the intent-to-treat analysis, Shafazanand et al. discovered a 3.1 mmHg decrease in mean BP. The study also notes that OSA therapy, especially when combined with three to four antihypertensive drugs, has an additive effect on BP control [18].

Degree of HTN: Hermida RC et al. discovered that OSA patients with high BP who were resistant to antihypertensive medication experienced a considerable drop in BP after using CPAP for almost a year. This allowed this group to go off their antihypertensive prescription therapy. Contrarily, CPAP did not cause a further decline in BP in the group of OSA patients whose BP was already under control with antihypertensives [16]. Additionally, Shirahama et al. proposed that, compared to normotensive subjects, hypertensive patients may have a more significant BP-lowering response to good CPAP adherence [17]. Hu et al. advocated CPAP with antihypertensive medication therapy as a recommended standard treatment for OSA and HTN [10]. According to Dernaika et al., a sizable fraction of patients with resistant HTN could reduce their antihypertensive medication dosage using CPAP therapy [10].

Limitations

The study has some limitations, including a scarcity of high-level evidence, for example, systemic reviews, randomized controlled trials, and meta-analyses. All of the studies identified were based on the limited number of clinical trials that were available. All of the studies showed variations in sample size and variable measurement. Not all of the studies examined had the same variables and secondary outcomes. This review included only papers written in English; thus, information from papers written in languages other than English was excluded. Animal studies were also excluded.

## Conclusions

We found evidence that CPAP therapy significantly lowers 24-hour ambulatory BP in those with severe OSA. The primary characteristic of OSA is the recurring partial or complete collapse of the upper airway during sleep. This collapse leads to intermittent episodes of reduced oxygen levels (hypoxemia) and disrupted sleep patterns. The development of higher inspiratory effort during hypopneas and apneas, repeated nocturnal hypoxia, fragmented sleep, or greater pleural pressure declines can all cause an increase in sympathetic tone. In addition to increasing ventilation and keeping the airway open, CPAP also lowers oxidative stress and stabilizes oxygenation, which suppresses vascular endothelial damage. Additionally, CPAP inhibits sympathetic nervous activity. OSA is regarded as a separate risk factor for cardiac rhythm problems, arterial HTN, and the onset and progression of diabetes. According to our research, in patients with severe OSA, BP response to CPAP treatment may be significantly influenced by obesity, hypersomnolence, high nocturnal BP, longer CPAP use, and resistant HTN. To assess the long-term impact of ventilation therapy in this population, more research is required. It would be particularly fascinating to evaluate the advantages of CPAP in a particular patient population that had just received a diagnosis of resistant HTN before the onset of irreversible CV impairment.

## References

[1] Benjafield AV, Ayas NT, Eastwood PR (2019). Estimation of the global prevalence and burden of obstructive sleep apnoea: a literature-based analysis. Lancet Respir Med.

[2] Yeghiazarians Y, Jneid H, Tietjens JR (2021). Obstructive sleep apnea and cardiovascular disease: a scientific statement from the American Heart Association. Circulation.

[3] Gunta SP, Jakulla RS, Ubaid A, Mohamed K, Bhat A, López-Candales A, Norgard N (2022). Obstructive sleep apnea and cardiovascular diseases: sad realities and untold truths regarding care of patients in 2022. Cardiovasc Ther.

[4] Rana D, Torrilus C, Ahmad W, Okam NA, Fatima T, Jahan N (2020). Obstructive sleep apnea and cardiovascular morbidities: a review article. Cureus.

[5] Weiss P, Kryger M (2016). Positive airway pressure therapy for obstructive sleep apnea. Otolaryngol Clin North Am.

[6] Bangash A, Wajid F, Poolacherla R, Mim FK, Rutkofsky IH (2020). Obstructive sleep apnea and hypertension: a review of the relationship and pathogenic association. Cureus.

[7] White DP (2016). Is continuous positive airway pressure an effective and practical antihypertensive agent?. Am J Respir Crit Care Med.

[8] Lai S, Mordenti M, Mangiulli M (2019). Resistant hypertension and obstructive sleep apnea syndrome in therapy with continuous positive airway pressure: evaluation of blood pressure, cardiovascular risk markers and exercise tolerance. Eur Rev Med Pharmacol Sci.

[9] Turnbull CD, Sen D, Kohler M, Petousi N, Stradling JR (2019). Effect of supplemental oxygen on blood pressure in obstructive sleep apnea (SOX). A randomized continuous positive airway pressure withdrawal trial. Am J Respir Crit Care Med.

[10] Hu X, Fan J, Chen S, Yin Y, Zrenner B (2015). The role of continuous positive airway pressure in blood pressure control for patients with obstructive sleep apnea and hypertension: a meta-analysis of randomized controlled trials. J Clin Hypertens (Greenwich).

[11] Liu L, Cao Q, Guo Z, Dai Q (2016). Continuous positive airway pressure in patients with obstructive sleep apnea and resistant hypertension: a meta-analysis of randomized controlled trials. J Clin Hypertens (Greenwich).

[12] Sova M, Sovova E, Hobzova M, Zapletalova J, Kamasova M, Kolek V (2015). The effect of continuous positive airway pressure therapy on the prevalence of masked hypertension in obstructive sleep apnea patients. Biomed Pap Med Fac Univ Palacky Olomouc Czech Repub.

[13] McEvoy RD, Michael MZ (2015). Measuring blood microRNAs to provide personalized advice to sleep apnea patients with resistant hypertension: dreaming the future. J Am Coll Cardiol.

[14] Litvin AY, Sukmarova ZN, Elfimova EM, Aksenova AV, Galitsin PV, Rogoza AN, Chazova IE (2013). Effects of CPAP on "vascular" risk factors in patients with obstructive sleep apnea and arterial hypertension. Vasc Health Risk Manag.

[15] Dernaika TA, Kinasewitz GT, Tawk MM (2009). Effects of nocturnal continuous positive airway pressure therapy in patients with resistant hypertension and obstructive sleep apnea. J Clin Sleep Med.

[16] Budhiraja R, Quan SF (2009). When is CPAP an antihypertensive in sleep apnea patients?. J Clin Sleep Med.

[17] Shirahama R, Tanigawa T, Ida Y (2021). Long-term effect of continuous positive airway pressure therapy on blood pressure in patients with obstructive sleep apnea. Sci Rep.

[18] Shafazand S, Patel SR (2014). Effect of CPAP on blood pressure in patients with obstructive sleep apnea and resistant hypertension. J Clin Sleep Med.

[19] Sánchez-de-la-Torre M, Khalyfa A, Sánchez-de-la-Torre A (2015). Precision medicine in patients with resistant hypertension and obstructive sleep apnea: blood pressure response to continuous positive airway pressure treatment. J Am Coll Cardiol.

[20] Castro-Grattoni AL, Torres G, Martínez-Alonso M (2017). Blood pressure response to CPAP treatment in subjects with obstructive sleep apnoea: the predictive value of 24-h ambulatory blood pressure monitoring. Eur Respir J.

[21] Bottini P, Taranto-Montemurro L, Novali M (2012). Effects of CPAP on systemic hypertension in OSAH: a monocentric, observational, cohort study. Respir Med.

[22] Ginosar Y, Malhotra A, Schwartz E (2013). High altitude, continuous positive airway pressure, and obstructive sleep apnea: subjective observations and objective data. High Alt Med Biol.

[23] Ogawa A, Emori T, Sumita W, Watanabe A, Fujio H, Miyaji K, Ohe T (2006). Continuous positive airway pressure ameliorated severe pulmonary hypertension associated with obstructive sleep apnea. Acta Med Okayama.

[24] Cardoso CR, Roderjan CN, Cavalcanti AH, Cortez AF, Muxfeldt ES, Salles GF (2020). Effects of continuous positive airway pressure treatment on aortic stiffness in patients with resistant hypertension and obstructive sleep apnea: a randomized controlled trial. J Sleep Res.

[25] Robinson GV, Smith DM, Langford BA, Davies RJ, Stradling JR (2006). Continuous positive airway pressure does not reduce blood pressure in nonsleepy hypertensive OSA patients. Eur Respir J.

[26] Del Pinto R, Grassi G, Ferri C (2021). Diagnostic and therapeutic approach to sleep disorders, high blood pressure and cardiovascular diseases: a consensus document by the Italian Society of Hypertension (SIIA). High Blood Press Cardiovasc Prev.

[27] Campos-Rodriguez F, Navarro-Soriano C, Reyes-Nuñez N (2019). Good long-term adherence to continuous positive airway pressure therapy in patients with resistant hypertension and sleep apnea. J Sleep Res.

